# AI-Facilitated Cognitive Reappraisal via Socrates 2.0: Mixed Methods Feasibility Study

**DOI:** 10.2196/80461

**Published:** 2025-12-05

**Authors:** Philip Held, Sarah A Pridgen, Daniel R Szoke, Yaozhong Chen, Zuhaib Akhtar, Darpan Amin

**Affiliations:** 1 Department of Psychiatry and Behavioral Sciences, Rush University Medical Center, 1645 West Jackson Boulevard, Suite 602, Chicago, IL, 60612, United States

**Keywords:** generative artificial intelligence, cognitive behavior therapy, digital mental health, large language models, cognitive reappraisal

## Abstract

**Background:**

Innovative, scalable mental health tools are needed to address systemic provider shortages and accessibility barriers. Large language model–based tools can provide real-time, tailored feedback to help users engage in cognitive reappraisal outside traditional therapy sessions. Socrates 2.0 (Rush University Medical Center) is a multiagent artificial intelligence tool that guides users through Socratic dialogue.

**Objective:**

The study aimed to examine the feasibility, acceptability, and potential for symptom reduction of Socrates 2.0.

**Methods:**

A total of 61 adult participants enrolled in a 4-week mixed methods preclinical feasibility study. The participants used Socrates 2.0 as desired and completed the self-report measures of depression, social anxiety, posttraumatic stress, and obsessive-compulsive symptoms at baseline and 1-month follow-up. Feasibility, acceptability, and appropriateness, along with usability and working alliance, were assessed via validated measures. The semistructured interviews explored user experiences and perceptions.

**Results:**

Participants engaged with Socrates 2.0 an average of 6.70 (SD 4.57) times over 4 weeks. Feasibility (mean 4.26, SD 0.67), acceptability (mean 4.16, SD 0.84), and usability ratings were high. Participants reported small-to-moderate reductions in depression (effect size *d*=0.30), social anxiety (*d*=0.25), obsessive-compulsive (*d*=0.33), and posttraumatic stress (*d*=0.28) symptoms. Working alliance scores suggested a moderately strong perceived bond with the artificial intelligence tool. Qualitative feedback indicated that the nonjudgmental, on-demand nature of Socrates 2.0 encouraged self-reflection and exploration. Some users critiqued the repeated questioning style and limited conversation depth.

**Conclusions:**

Socrates 2.0 was perceived as feasible, acceptable, and moderately helpful for self-guided cognitive reappraisal, demonstrating potential as an adjunct to traditional therapy. Further research, including randomized trials, is needed to determine effectiveness across different populations, optimize personalization, and address the repetitive conversational nature.

## Introduction

Despite the growing demand for mental health services, access to care continues to be limited by several barriers, such as provider shortages, logistical constraints such as time and travel, financial burden, and perceived stigma surrounding therapy [1,2]. Digital mental health interventions offer promising avenues to deliver support, overcome wait times, and meet a wide variety of patient preferences [1,2]. Traditional applications designed for cognitive behavioral therapy (CBT) have primarily relied on rule-based logic and responses, which has led to interactions that can feel impersonal and static [3]. Recent advancements in artificial intelligence (AI), particularly in large language models (LLMs), can potentially address these limitations by providing human-like, context-sensitive responses [4-6]. Numerous LLM-based therapy tools are currently in development [7], but rigorous safety and feasibility data are needed before such tools can be widely adopted [4,5].

One CBT technique shown to be effective in achieving cognitive reappraisal is the Socratic dialogue, which invites patients to systematically examine and, when appropriate, revise maladaptive beliefs [8]. Outside of therapy sessions, patients are assigned to complete worksheets designed to facilitate similar cognitive reappraisal processes [9,10]. However, worksheets lack the interactivity and immediate feedback patients may need to remain engaged and may at least partially explain the high homework noncompletion rates that have been observed [11,12]. LLM-based digital tools that emulate therapists’ Socratic dialogue style may be more engaging than static worksheets and thereby increase adherence to out-of-session practice and ultimately help users engage in cognitive reappraisal exercises [13].

Socrates 2.0 (Rush University Medical Center) is an LLM-based tool developed to engage users in Socratic dialogue. The tool leverages Microsoft Azure’s OpenAI’s GPT-4o model. Prompt engineering was led by clinical psychologists with expertise in cognitive therapy, including Socratic questioning and cognitive restructuring. An LLM-based tool differs greatly from traditional rule-based CBT apps that rely on predetermined answers that are given verbatim after prespecified user inputs. Socrates 2.0 generates unique, context-sensitive responses based on the user’s input. Rather than a single AI chatbot, such as ChatGPT, Socrates 2.0 uses a multi-AI agent framework. Three distinct AI agents interact directly with one another to engage the user in Socratic dialogue. The user only interacts directly with an AI therapist. Hidden from the user, an AI supervisor monitors the entire dialogue and provides the AI therapist with concrete suggestions for how to improve the Socratic dialogue and the overall experience of the user. Also hidden from the user, an AI rater evaluates changes in the users’ belief as an indicator of possible progress and provides this information to the AI therapist so it can adjust the course of the intervention as needed. Compared to an earlier single-agent version of the tool, the addition of the 2 supervisory AI agents appears to improve dialogue quality by reducing the risk of redundant, off-topic, or overly lengthy answers and repetitive conversational loops (ie, segments in which similar questions or statements are repeated without progressing the dialogue) [13]. One example of a conversational loop-out that the team encountered while using Socrates 1.0, an earlier version that did not include supervisory AI agents, is that an AI therapist would ask about evidence for and against a belief. When a user provided evidence for and against a belief, the AI therapist would sometimes repeat the question about evidence for and against a belief, even when this information was recently provided (ie, looping). These issues were identified through both user testing and transcript review conducted by the research team. The additional AI agents also help maintain focus and intervene if dialogues shift toward inappropriate or harmful directions. Development followed an iterative process of testing and refinement, in which prompts and role instructions were repeatedly adjusted to enhance fidelity to Socratic technique. More information about the development of Socrates 2.0 can be found in a study by Held et al [13].

A prior companion study examined clinician perceptions of Socrates 2.0 to evaluate therapeutic appropriateness and safety during development [14]. Clinicians rated Socrates 2.0 as generally acceptable and feasible and saw potential for supplementing therapy, particularly for out-of-session cognitive reappraisal exercises. Clinicians remarked that the AI interface could make conventional CBT homework feel more approachable and interactive [14], but data from end users (eg, patients) are needed. This study was conducted as a distinct follow-up phase to assess end-user feasibility, acceptability, and perceived benefit once clinician input had informed model refinements.

In this feasibility study, we examined (1) how users interacted with the Socrates 2.0 when practicing Socratic dialogue independently, (2) whether they perceived it as accessible, helpful, and safe, (3) the frequency and duration of their engagements, and (4) changes in anxiety, depression, posttraumatic stress, psychosis, obsessive-compulsive, and mania-related symptom measures.

## Methods

### Eligibility, Recruitment, and Participants

Individuals were eligible to participate in this study if they were at least aged 18 years, were able to read and write in English at a sixth-grade level or higher, had access to an internet-capable device (ie, computer, tablet, or smartphone), and were potentially interested in using a self-guided online mental health resource. Because the study was preclinical and designed to examine broad feasibility and acceptability, no specific mental health diagnosis or current treatment was required, and no exclusions were applied beyond the listed inclusion criteria. Participants were recruited via social media and word of mouth.

### Ethical Considerations

All study procedures were reviewed and approved by the Rush University Medical Center Institutional Review Board (IRB #23083108). Prior to participation in any study procedures, participants provided electronic informed consent. Participants were compensated up to US $50 in the form of electronic gift cards for their completion of study-related assessments and interviews.

### Procedures

#### Baseline Assessment

Following the consent process and prior to engaging with Socrates 2.0, participants electronically self-reported demographic characteristics. Participants were also asked to complete symptom measures including a validated brief version of common mental health instruments, which included the Patient Health Questionnaire-4 [15], Interaction Anxiousness Scale-3 (IAS-3) [16], Posttraumatic Stress Disorder (PTSD) Checklist-4 [17], Obsessive-Compulsive Inventory-4 [18], and Washington Early Recognition Center Affectivity and Psychosis Screen [19], and asked to participate in a short semistructured interview that assessed for their familiarity with AI in mental health care.

Established clinical cut-off scores indicating probable clinical range are approximately ≥6 on the Patient Health Questionnaire-4 for depression or anxiety [15], ≥10 on the IAS-3 for social anxiety [16], ≥4 on the PTSD Checklist-4 for PTSD [20], and ≥4 on the Obsessive-Compulsive Inventory-4 for obsessive-compulsive symptoms [21]. A score of ≥13 on the Washington Early Recognition Center Affectivity and Psychosis Screen [22] has been suggested for potential mood or psychosis. The IAS-3 does not have an established cut-off; scores are summed from 3*=least anxious* to 15*=most anxious*. Mean baseline scores in this sample were often below these thresholds, consistent with a nonclinical but psychologically engaged population.

Participants were then introduced to Socrates 2.0 by a member of the study team. The introduction involved a general overview of the tool as well as a brief demonstration of the tool. During this time, the study team member answered any relevant questions. Participants were given secure log-in credentials for Socrates 2.0 and had free, unlimited access for 4 weeks. They were encouraged to log in at any time and as often as they wished to examine their beliefs or thoughts. As described in a study by Held et al [13], the AI interface also displayed safety notices, reminding participants of the nonclinical nature of the tool and providing information on how to seek professional help if needed.

#### One-Month Assessment

Participants repeated the same symptom measures after the 4-week study period and also completed a set of validated usability and acceptability measures, such as the System Usability Scale [23], the Acceptability of Intervention Measure, the Intervention Appropriateness Measure, the Feasibility of Intervention Measure [24], and the mHealth App Usability Questionnaire [25]. Participants were also asked to complete the Working Alliance Inventory–Short Revised [26] to evaluate whether they experienced any sense of collaborative alliance with the AI system, a validated assessment traditionally associated with measuring therapeutic rapport. Finally, each participant took part in a semistructured interview to obtain detailed feedback on their overall impressions of Socrates 2.0, areas they found most beneficial, perceived shortcomings, use behaviors, and suggestions for future refinements.

### Data Analysis

Quantitative analyses were conducted using R (version 4.5.1; R Foundation for Statistical Computing). Paired-sample 2-tailed *t* tests were used to compare baseline and 1-month scores across outcome measures. Given the exploratory feasibility design, adjustments for multiple comparisons were not applied. Effect sizes (Cohen *d*) were calculated to estimate the magnitude of change.

Qualitative responses were analyzed using thematic analysis [27], informed by principles of Consensual Qualitative Research to minimize individual bias [28]. Two mental health researchers independently reviewed all participant responses to become familiar with the data and noted initial impressions. Each then independently generated preliminary codes to capture salient ideas, which were discussed and refined through consensus meetings to develop a shared codebook. Related codes were grouped into broader themes that reflected recurring patterns across responses. The team collaboratively defined and named these themes, selected representative quotations, and organized the results to illustrate participants’ experiences with Socrates 2.0.

## Results

### Overview

A total of 61 participants were involved in the study, which included male (32/61, 52%), non-Hispanic (51/61, 84%), and White participants (32/61, 52%), and the average age was 39.9 (SD 15.50) years. Less than half (29/61, 48%) expressed having working knowledge of AI tools, particularly Open AI’s ChatGPT, Google Gemini, and SnapchatAI. Approximately 74% (45/61) reported having been in therapy at some point. Further demographic information can be found in Table 1. On average, participants engaged with Socrates 6.70 (SD 4.57) times and averaged 10.44 (SD 7.65) exchanges per conversation. Feasibility (Feasibility of Intervention Measure: mean 4.26, SD 0.67), acceptability (Acceptability of Intervention Measure: mean 4.16, SD 0.84), and implementation (Intervention Appropriateness Measure: mean 4.13, SD 0.91) were rated highly, as was usability (System Usability Scale and mHealth App Usability Questionnaire results can be found in Table 2). Participants reported small-to-moderate changes in many mental health symptoms and rated the working alliance with Socrates 2.0 to be moderately strong (Table 3). Below is the analyzed participant feedback, categorized by positive, negative, and improvement-oriented feedback, as well as general comments.

**Table 1.** Characteristics (N=61).

Variable	Value
Gender, n (%)	
Woman	28 (46)
Man	32 (52)
Other	1 (2)
Age (y), mean (SD)	39.9 (15.50)
Race, n (%)	
Asian	5 (8)
Black or African American	17 (28)
Other	2 (3)
White	32 (53)
No response	5 (8)
Ethnicity	
Hispanic or Latino	7 (11)
Not Hispanic or Latino	51 (84)
No response	3 (5)
Education level	
High school diploma	6 (10)
Trade or technical college	8 (13)
Associate degree	4 (7)
Bachelor’s degree	26 (43)
Master’s degree	15 (25)
Doctoral degree	2 (3)

**Table 2.** Satisfaction questionnaire results.

Questionnaire	Value
SUS^a^ item	
Agree or strongly agree (%)	
I think that I would like to use Socrates 2.0 frequently.	73.77
I thought Socrates 2.0 was easy to use.	91.80
I found the various functions in Socrates 2.0 were well integrated.	70.49
I would imagine that most people would learn to use Socrates 2.0 very quickly.	81.97
I felt very confident using Socrates 2.0.	80.33
Disagree or strongly disagree (%)	
I found Socrates 2.0 unnecessarily complex.	88.52
I think that I would need the support of a technical person to be able to use Socrates 2.0.	93.44
I thought there was too much inconsistency in Socrates 2.0.	90.16
I found Socrates 2.0 very cumbersome to use.	75.41
I needed to learn a lot of things before I could get going with Socrates 2.0.	90.16
MAUQ^b^	
Agree or strongly agree (%)	
Socrates 2.0 was easy to use.	96.72
It was easy for me to learn to use Socrates 2.0.	96.72
The navigation was consistent when moving between screens.	81.96
The interface of Socrates 2.0 allowed me to use all the functions offered by Socrates.	70.49
Whenever I made a mistake using Socrates 2.0, I could recover easily and quickly.	68.85
I like the interface of Socrates 2.0.	68.85
The information in Socrates 2.0 was well organized, so I could easily find the information I needed.	78.69
Socrates 2.0 adequately acknowledged and provided information to let me know the progress of my action.	72.13
I feel comfortable using Socrates 2.0 in social settings.	63.93
The amount of time involved in using Socrates 2.0 has been fitting for me.	75.41
I would use Socrates 2.0 again.	86.89
Overall, I am satisfied with Socrates 2.0.	85.25
Socrates 2.0 would be useful for my health and well-being.	78.69
Socrates 2.0 improved my access to health care services.	44.26
Socrates 2.0 helps me manage my health effectively.	62.29
Socrates 2.0 has all the functions and capabilities I expected it to have.	65.57
I could use Socrates 2.0 even when the internet connection was poor or not available.	32.79
Socrates 2.0 provided an acceptable way to receive health care services, such as accessing education materials, tracking my own activities, and performing self-assessment.	29.51

a SUS: System Usability Scale.

b MAUQ: mHealth App Usability Questionnaire.

**Table 3.** Working alliance and mental health symptom change.

Measure (condition; possible range)	Baseline, mean (SD)	1-month assessment (after), mean (SD)	Change, mean (SD*)*	Significance (*P* value)	Effect size (Cohen *d*)
PCL-4 (PTSD^b^; 0‐16)	5.89 (4.27)	4.66 (4.41)	1.23 (3.25)	.004	0.28
PHQ-4^c^ (depression; 0‐12)	4.02 (3.03)	3.13 (2.93)	0.89 (2.22)	.002	0.30
IAS-3^d^ (social anxiety; 3‐15)	8.20 (3.45)	7.33 (3.46)	0.87 (2.54)	.009	0.25
OCI-4^e^ (obsessive compulsive traits; 0‐16)	4.28 (3.92)	3.07 (3.37)	1.21 (2.71)	.001	0.33
WERCAPS^f^ (mood and psychosis; 0‐25)	4.30 (4.74)	3.74 (4.76)	0.56 (3.01)	.153	0.12
WAI-SR^g^ (agreement on tasks; 4-20)	N/A^h^	14.21 (4.68)	N/A	N/A	N/A
WAI-SR (agreement on goals; 4-20)	N/A	13.67 (4.70)	N/A	N/A	N/A
WAI-SR (bond; 4-20)	N/A	13.69 (5.02)	N/A	N/A	N/A

a PCL-4: Posttraumatic Stress Disorder Checklist-4.

b PTSD: posttraumatic stress disorder.

c PHQ-4: Patient Health Questionnaire-4.

d IAS-3: Interaction Anxiousness Scale-3.

e OCI-4: Obsessive-Compulsive Inventory-4.

f WERCAPS: Washington Early Recognition Center Affectivity and Psychosis Screen.

g WAI-SR: Working Alliance Inventory–Short Revised.

h N/A: not applicable.

### Positive Themes

#### Encourages Reflection and Self-Discovery

Many participants described Socrates 2.0 as prompting them to explore issues and consider perspectives they had not previously entertained. The tool’s AI-driven questions appeared to help users uncover negative thought patterns and cognitive “blind spots.”

*It helped me explore ideas and thoughts… helpful to anyone on a journey of finding out why you’re stuck or what keeps you in one particular place.*

[SOC088]

*The questions made me think differently about some problems I was stuck on.*

[SOC063]

A few participants remarked that the tool pushed them to confront core beliefs they had never verbalized, resulting in new insights and opportunities for personal growth.

*It got me to think about things I hadn’t considered before.*

[SOC075]

*It helped me process issues I wasn’t even aware I had.*

[SOC035]

*The tool prompted me to dig deeper into my thoughts and explore underlying issues.*

[SOC090]

These accounts underscore Socrates 2.0’s potential as a springboard for greater self-awareness, even if further support or guidance from a human professional may be required for more serious concerns.

#### Nonjudgmental Interactions

Another salient theme was the sense of safety and comfort users experienced, which most attributed to the absence of perceived judgment from an AI. Participants reported feeling more at ease discussing sensitive topics or admitting certain vulnerabilities they might hesitate to share with a human therapist.

*It felt like a safe space, free of judgment, which was comforting.*

[SOC111]

*I really like Socrates. It’s really like warm and welcoming. I like the language that it uses. It’s neutral and talks in an accepting way. You don’t feel judged at all, which I think is extremely important when it comes to any type of mental health tool.*

[SOC007]

*It should be an adjunct or something that that kind of preps you and warms you for going into therapy or for other mental health help.*

[SOC035]

Several individuals contrasted these interactions with past experiences of stigma or fear of judgment in therapeutic settings.

*I just wanted to have a conversation with someone who wouldn’t be judgmental.*

[SOC055]

*[Socrates has] no judgement. Maybe there are some things you wouldn't feel comfortable talking to someone [about]. It would be good for things like that.*

[SOC003]

*I'm a private person. I love the aspect of it not being a real person.*

[SOC007]

#### Potential Benefit for Mental Health

Several participants suggested that Socrates 2.0 could serve as a “first line” resource or adjunct to formal therapy, particularly for those unable to regularly attend appointments.

*It can be seen as a first response or something to combine with therapy… something you can use at home or on the go.*

[SOC116]

One individual contrasted it with general LLM-based tools, noting the Socrates’ mental health specificity:

*This doesn’t work like ChatGPT at all. This is more emotional… It kind of understands humans better.*

[SOC007]

Many participants praised the option to access Socrates 2.0 at any time of day, highlighting the advantage of self-directed sessions without the need to schedule formal therapy appointments.

*I liked that I could use it anytime I wanted, without needing to wait for a therapist.*

[SOC063]

*Being able to access it on my phone was convenient and easy to navigate.*

[SOC113]

Finally, some individuals found that, after some time using Socrates 2.0, they had learned how to engage in the Socratic process without needing the tool.

*I’ve started applying what it’s spitting back to may. And maybe it actually has influenced the way I think because now I don’t even feel like I need to go on the app. Like, if I feel like I need to go on the app, I’m like okay, this is what it’s going to tell me anyway. Maybe I should just think about it this way.*

[SOC003]

### Negative Themes

#### Repetitive Nature

Although iterative questioning is a hallmark of Socratic dialogue, numerous participants felt the responses were too similar, which was experienced as tedious and reduced participants’ desire to continue interactions.

*The interactions felt repetitive at times, which reduced its overall effectiveness.*

[SOC051]

*Instead of giving me straight answers to my questions, it kept asking me questions on my questions.*

[SOC055]

#### Lack of Depth

A subset of users felt that Socrates 2.0 was unable to thoroughly address complex emotional concerns or trauma-related issues, describing some responses as superficial or generic.

*Some of the responses felt surface-level and didn’t address deeper concerns.*

[SOC049]

*It doesn’t always account for the complexity of human emotions.*

[SOC043]

Several participants suggested that although the tool was helpful for prompting initial self-reflection, it might not be adequate for more severe mental health challenges.

### Suggested Improvements

#### Overview

In light of these negative aspects, participants offered constructive suggestions.

#### Dynamic and Diverse Questioning

Users advocated for the inclusion of more varied prompts to prevent repetitive conversational loops. They also proposed that the AI should adapt questions as it learns from each user’s personal context.

*Adding more variation in the types of questions would keep the conversations engaging.*

[SOC085]

*Adding a mechanism to avoid repetitive loops in the conversation would help.*

[SOC128]

#### Mobile App Development and User Interface

Though many accessed Socrates 2.0 on their smartphones via a web browser, participants felt a dedicated mobile app could simplify the user experience. Additional suggestions included improved navigation and frictionless login.

*An app would make it more convenient and encourage greater usage.*

[SOC132]

*A Remember Me button would make it easier to log in without typing credentials repeatedly.*

[SOC031]

#### Enhanced Personalization

Finally, participants expressed that the tool would benefit from remembering previous conversation threads, tailoring questions, and offering more individualized follow-up.

*If it could remember past interactions and adapt, it would feel more personal.*

[SOC126]

*Making the system more responsive to individual differences would be beneficial.*

[SOC097]

## Discussion

This study evaluated the feasibility and acceptability of Socrates 2.0, a multiagent AI tool designed to engage individuals in Socratic dialogue. Participants generally reported high levels of satisfaction with Socrates 2.0’s accessibility and a nonjudgmental interaction style. In line with other digital mental health tools, it appears that the 24/7 access can be a key factor for high user engagement, particularly among those who avoid traditional in-person therapy due to logistical constraints or concerns about stigma [4,29,30]. In this study, participants described Socrates 2.0 as a safe space to explore personal struggles. Some participants even reported disclosing things to the tool that they would have been hesitant to discuss with a human therapist for fear of judgment. In other cases, participants remarked that working with Socrates 2.0 was a way for them to practice what to say to their human therapist at a later time. These insights contrast common fears associated with the use of AI in the context of mental health care, where privacy is commonly listed as a key issue [5,6]. Notably, none of the participants in this study raised privacy or ethical concerns regarding their use of Socrates 2.0. This may have been due to the detailed informed consent process, which clearly explained the data-handling procedures, including where data were stored and who had access to the data, and emphasized that the tool was not a substitute for therapy. Although self-selection may be a key factor, the absence of reported concerns may also suggest that transparency around privacy protocols is important when engaging with AI-based mental health tools.

Many participants praised the human-like quality of Socrates 2.0’s responses, which was further supported by above-average ratings of the working alliance. It is plausible that the human-like responses that LLMs produce lead to a greater sense of being understood compared to rule-based algorithms where responses often did not feel tailored to the individual [3]. To optimize future tools, research should compare theorized therapeutic mechanisms, such as the working alliance [31], between algorithms to identify the exact mechanisms that contribute to positive experiences and treatment outcomes. Interestingly, some individuals in our sample initially expected Socrates 2.0 to function like a general-purpose LLMs, such as ChatGPT, but found Socrates 2.0’s focus on cognitive reappraisal offered more depth and made exchanges more focused and meaningful. These findings support literature suggesting that specialized AI tools, rather than broad all-purpose models, may be more successful at engaging users in structured therapeutic exercises [4,7,32].

Participants also described several areas for improvement. In contrast with the positive quantitative and qualitative feedback, some participants described interactions as repetitive and said that they felt caught in circular conversations or that they were receiving basic follow-up questions instead of genuinely novel insights. Others remarked that conversations lacked depth at times. This perception likely reflects the inherent structure of Socratic dialogue, which depends on a sequence of open-ended, reflective questions intended to guide users towards deeper cognitive reappraisal rather than to provide direct suggestions or advice. While this repetition served a therapeutic purpose, it may contrast with user expectations of conversational variety typically associated with general-purpose language models (eg, Open AI’s ChatGPT). In line with this, some users who had these criticisms remarked that they had hoped for specific advice and guidance from the AI therapist rather than continued questions. Another element that may have contributed to the repetitive nature is the supervisory agents within Socrates 2.0, which are designed to monitor the dialogue and ensure that the AI therapist maintains focus on the user’s stated belief. This keeps the conversation on task and aligned with Socratic principles, but may also increase what some users perceive as repetitive statements when users attempt to shift topics or seek broader discussion. In addition to refining the hidden supervisory AI agents, it will be important to evaluate the impact of different AI therapist response styles as well as the importance of setting expectations for the interactions with tools like Socrates 2.0. This may be especially relevant for individuals who have firm ideas about what therapy should look and feel like and expect interactions with an AI therapist to mimic such expectations. It may also be important to leverage LLMs to detect possible conversational fatigue and build an adaptive algorithm that uses such data to signal to the AI therapist to adjust the response style.

Although accessibility was a key reason for developing Socrates 2.0, only a subset of participants endorsed survey items reflecting improvement in access to care. Possible reasons for this may include items being worded too broadly, such as by asking about health care access, whereas Socrates 2.0 was only designed to facilitate one concrete therapeutic task rather than function as an alternative to mental healthcare or even health care as a whole. At the same time, the tested version of Socrates 2.0 had some accessibility limitations that may have contributed to the lower survey item endorsement. For example, the tested version was designed to be used on a desktop or laptop and was not optimized for use on smartphones. In addition, this study required users to log in manually with a pregenerated username and password, which may have impacted the perceived convenience. Future iterations of the tool have already begun to address accessibility limitations through streamlining login procedures for users and providing a better mobile experience.

As part of this study, we also gathered descriptive data on how frequently and when participants chose to interact with Socrates 2.0. Whereas some participants reported logging in for short “bursts” of conversation during moments of stress or uncertainty, others preferred extended sessions in the evenings or weekends, which are perhaps more akin to traditional therapy appointments. These data suggest that individuals use digital mental health tools differently compared to traditional 50-minute therapy appointments and underscore the need for such tools to be designed differently than to simply mimic human-delivered therapy sessions.

Despite promising indications, the study carries notable limitations that shape the interpretation and applicability of its findings. First, participants self-selected into the study and may have been more comfortable with technology and AI than the general population. Second, the feasibility-focused design lacked a control or comparison group and only included exploratory quantitative analyses; therefore, the significance of these findings is limited. Although participants reported a range of mental health symptoms, the sample was not selected based on clinical diagnoses, and symptom severity was generally low across all screening measures. Future research should assess feasibility, acceptability, and symptom impact in clinical populations with confirmed mental health diagnoses. The sample was also highly educated, which may limit generalizability to broader populations. Though LLMs are flexible at accommodating a range of reading levels and communication styles, future research should evaluate the tools' usability and acceptability across more diverse educational and demographic backgrounds. Finally, digital literacy was not systematically assessed and may have influenced the frequency and effectiveness of user engagement with Socrates 2.0.

Socrates 2.0 holds clear potential to facilitate cognitive reappraisal outside of therapy sessions, largely due to its 24/7 availability, sense of anonymity, and engaging conversational style. Participants’ remarks often framed Socrates 2.0 as a potential adjunct rather than a replacement for human-delivered therapy, which reflects a stance increasingly recommended in the literature: AI tools may be best used within a stepped-care or blended-care model, where AI-driven conversation augments but does not supplant clinician-led treatment [4]. Future research should examine multi-month engagement patterns and involve randomized controlled trials comparing Socrates 2.0 with standard CBT worksheets to determine whether the AI-driven dialogue can produce measurable clinical advantages. Research should also examine different subgroups, including participants with severe symptoms, culturally diverse backgrounds, or varying degrees of digital literacy. Overall, this work underscores the promise and challenges of LLM-driven tools in the domain of mental health and highlights an important and exciting avenue for future research and development as AI technologies continue to evolve.

## Abbreviations

AI: artificial intelligence
CBT: cognitive behavioral therapy
IAS-3: Interaction Anxiousness Scale-3
LLM: large language model
PTSD: posttraumatic stress disorder
